# Clinical neuropathology of early‐stage Pick's disease initially diagnosed as depressive disorder: A case report and case series comparison

**DOI:** 10.1002/pcn5.70116

**Published:** 2025-05-08

**Authors:** Araki Kimura, Ito Kawakami, Kenji Ikeda, Akito Nagakura, Kazuhiro Niizato, Kenichi Oshima, Tadafumi Kato, Masato Hasegawa

**Affiliations:** ^1^ Dementia Research Project Tokyo Metropolitan Institute of Medical Science Setagaya Tokyo Japan; ^2^ Department of Psychiatry and Behavioral science Juntendo University Graduate School of Medicine Bunkyo Tokyo Japan; ^3^ Department of Psychiatry Tokyo Metropolitan Matsuzawa Hospital Setagaya Tokyo Japan; ^4^ Division of Molecular Pathology and Histology Tokyo Metropolitan Institute of Medical Science Setagaya Tokyo Japan

**Keywords:** apathy, depression, pathology, Pick's disease, tau

## Abstract

**Background:**

Pick's disease (PiD) is a subtype of frontotemporal lobar degeneration. However, the pathogenesis and symptomatic lesions remain unclear. We report a case of PiD with a short disease duration and compare it to a case series to reveal the association between degenerative patterns and clinical manifestations.

**Case Presentation:**

The patient showed a marked decline in motivation at the age of 54 years. He was admitted with a clinical diagnosis of depressive disorder at the age of 56 years. He exhibited only apathy and lacked typical behavioral symptoms. Specialist observation revealed behavioral symptoms such as disinhibition, a lack of empathy, and hyperorality that had previously unnoticed by the patient's family members. The patient died of acute heart failure 4 days after hospitalization. Postmortem examination revealed a brain weight of 1090 g, with focal atrophy of the bilateral frontal and temporal lobes. Neuropathological findings mainly presented as numerous Pick bodies (PBs), mainly in the frontal lobe and hippocampus. PBs were immunopositive for phosphorylated tau and 3‐repeat tau but negative for 4‐repeat tau. The pathological findings in this case corresponded to phase II of PiD staging as defined in a previous study.

**Conclusion:**

The clinical symptoms in this case, primarily characterized by apathy with minimal behavioral symptoms, were consistent with the predominant pathological involvement of the dorsolateral frontal lobe. The present case was interpreted as early‐phase PiD. A comparison of the case series suggested that early‐phase PiD cases may help clarify the association between early clinical manifestations and focal degenerative lesions in the frontal lobe.

## Graphical abstract

This case of a 56‐year‐old patient who presented with apathy and few typical behavioral symptoms was initially diagnosed with depressive disorder. Postmortem examination revealed numerous Pick bodies (which were immunopositive for phosphorylated tau and 3‐repeat tau but negative for 4‐repeat tau), focal atrophy in the frontal lobes, and resulted in a pathological diagnosis of Pick's disease, frontotemporal lobar degeneration‐tau with 3‐repeat tau. The clinical symptoms, which were mainly apathy, were consistent with the pathological lesion that was dominantly in the dorsolateral frontal lobe.

## BACKGROUND

Pick's disease (PiD) is a subtype of frontotemporal lobar degeneration (FTLD).^1^ The clinical subtypes of FTLD are behavioral variant frontotemporal dementia (bvFTD), nonfluent variant primary progressive aphasia (nfvPPA), semantic variant primary progressive aphasia, and so forth. FTLD neuropathologically includes several types of tauopathies (FTLD‐tau) and the other proteinopathies. PiD accounts for ∼15% of all FTLD cases.^2^, ^3^, ^4^ PiD mostly presents with clinical subtypes, such as bvFTD, and some cases show nfvPPA.^5^ Patients with bvFTD show disinhibition, apathy, loss of empathy, stereotyped behavior, and social–emotional dysfunction.^6^ PiD presents focal atrophy of the frontal and temporal lobes and is histologically characterized by the formation of argyrophilic, round intraneuronal cytoplasmic inclusions (NCIs) called Pick bodies (PBs).^7^, ^8^, ^9^ PBs consist of phosphorylated 3‐repeat tau.^10^, ^11^, ^12^ The average disease duration of PiD cases is approximately 7 years.^13^, ^14^ Only a few studies have reported PiD cases with short disease durations,^15^, ^16^, ^17^ and with a prodromal state.^18^, ^19^, ^20^ Clinicopathological studies of early PiD are important for clarifying its propagation. This study provides insight into the clinical symptoms associated with histopathological lesions. Herein, we report a detailed clinical and neuropathological evaluation of a case of PiD with a short duration of 1.5 years after onset. Furthermore, we compared the representative case with a case series of PiD to elucidate which case, characterized by disease duration and the phase of neurodegeneration, has the potential to clarify the association between early clinical manifestations and degenerative lesions in the frontal lobe.

## CASE PRESENTATION

This case was previously reported in a case series^21^ assessing the distribution and propagation of degenerative lesions in PiD. In this report, we have added the immunohistochemical findings and a detailed history of the disease.

### Clinical information in the present case

The case is a male who died at the age of 56 years. Although not talkative, he was a reliable and upright person by nature. He was diligent and talented in his job. At the age of 54 years, 1.5 years before his death, he suddenly stopped going to work, showed a marked decline in motivation, and became unresponsive, even to his family. He spent most of his time in bed and was incontinent. He started to eat less and lost 6 kg. At the age of 56 years, he was admitted to a psychiatric hospital with a clinical diagnosis of depressive disorder. Neurological and blood tests revealed no abnormalities. Hasegawa's Dementia Scale showed impairments in attention and calculation, while orientation and short‐term nonverbal memory were preserved (18/32.5 points). According to the medical record, brain CT revealed bilateral frontal lobe atrophy. During hospitalization, the patient showed disinhibition and a lack of empathy as he walked away without any notification when a physician performed consultation or neuropsychological tests. He also showed decreased interest in himself and his surroundings, and hyperorality. He died of acute heart failure at the age of 56 years, 4 days after hospitalization.

### Material and methods

**Figure 1:**
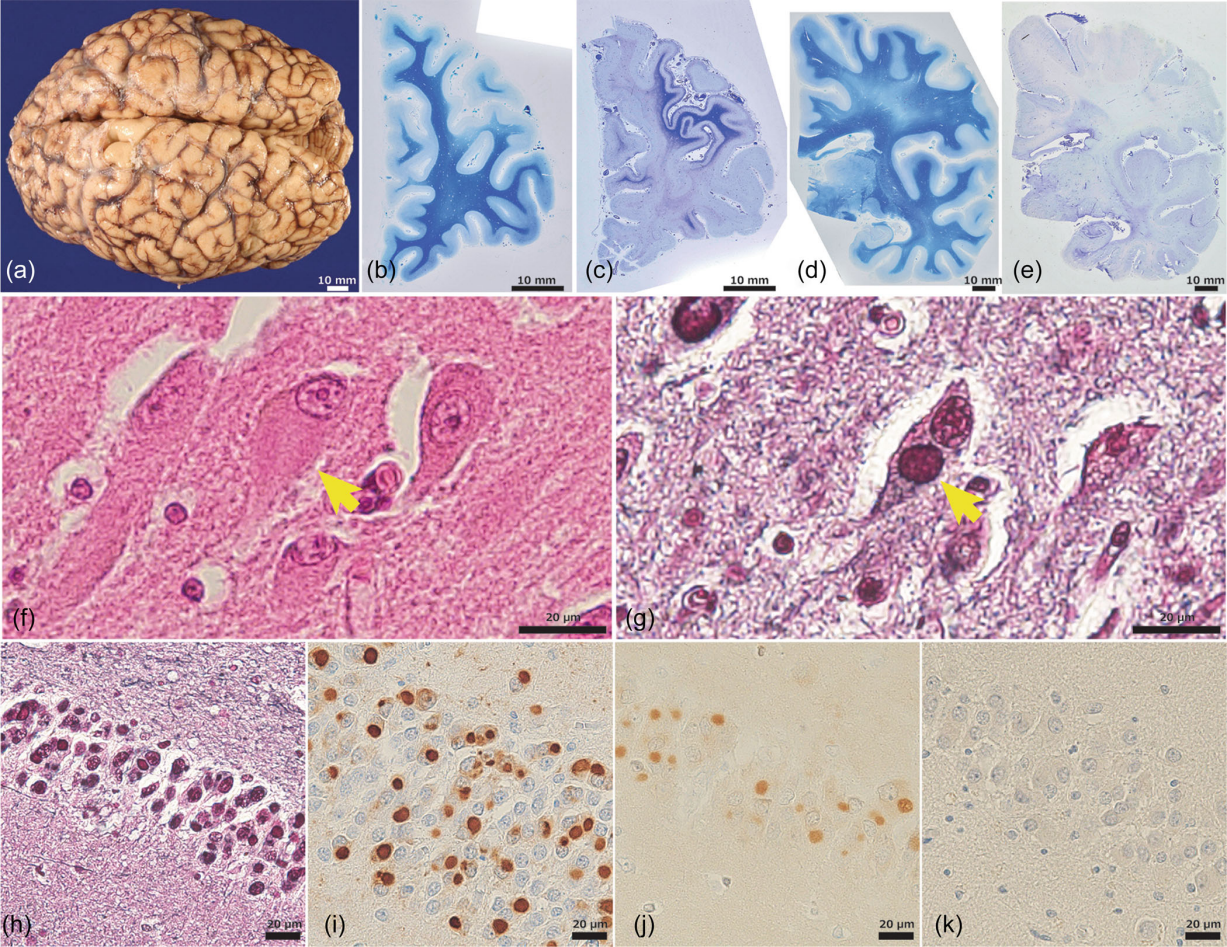
Gross and microscopic findings of the present case. (a) Mild atrophy of both frontal lobes is pronounced. Coronal section of the left hemisphere shows a (b) moderate frontal lobe and (d) slight medial temporal lobe atrophy. Holzer staining indicated (c) lateral surface dominant frontal lobe and (e) medial temporal lobe gliosis. Pick bodies in CA1 stained with (f) hematoxylin and eosin (H&E) and (g) Bodian stain. Pick bodies in dentate gyrus (DG) stained with (h) Bodian stain or immunolabeled with (i) AT8: anti‐phosphorylated tau and (j) anti‐3‐repeat tau (RD3): RD3. (k) Intraneuronal inclusions were not labelled with RD4: anti‐4‐repeat tau. The following primary antibodies were used for immunostaining: anti‐phosphorylated tau (AT8), RD3, anti‐4‐repeat tau (RD4), anti‐phosphorylated‐α‐synuclein (pSyn#64), and anti‐phosphorylated‐TDP 43 (pser409‐410). Immunostaining labelling was visualized by avidin‐biotinylated horseradish peroxidase complex (ABC) method (Vectastain Elite Kit; Vector Laboratories).

The autopsied brain was fixed in formaldehyde and sectioned along the coronal plane. The tissues were paraffin‐embedded and sliced at a thickness of 8 µm. For histopathological assessment, paraffin‐embedded slices were stained with hematoxylin and eosin (H&E), Klüver–Barrera (KB), Holzer, and Bodian stains, and were assessed by immunostaining as shown in Figure 1.

In addition, the distribution of lesions in phosphorylated tau‐positive structures was assessed in 11 patients with PiD registered in the neuropathological archives of the Tokyo Metropolitan Institute of Medicine to compare with the present case. The observation areas were determined with reference to a previous study^17^ to identify the propagated areas of phosphorylated tau‐positive structures, including both NCIs and degenerative neurites (Table 1). Pathological phases were defined according to a previous study (Table 1). In addition, we compared affected lobes of each case by H&E staining (Table 1). This PiD series included previously reported cases.^21^, ^22^, ^23^

**Table 1:** Pathological distribution of phosphorylated tau‐positive structures, including intraneuronal inclusion and degenerative neurites, and clinical information of each PiD case.

Patient number	Clinical phenotype	Initial symptoms	Age at onset (years)	Age at death (years)	Disease duration (years)	Sex	Brain weight (g)	Comparison of lesion severity in involved lobes	Phase^a^	Substantia nigra	Locus coeruleus	Dorsal nucleus of the vagus nerve	Arcuate nucleus	Hypoglossal nucleus	Visual cortex	Cerebellum granular layer	Cerebral peduncle
1 (present case)^b^	bvFTD	Apathy	54	56	1.5	M	1090	F(l > c > o)>T(m = i > s)>P	II	2	2	1	0	0	0	0	0
2^b^	nfvPPA, Unspecified dementia	Dyslexia, Stereotypic behavior	48	56	7	F	950	F(o = c > l)≥T(m = i > s)>P	III	—	2	2	2	0	0	0	—
3^c^	Apraxia of speech, Sensory aphasia, Unspecified dementia	Memory impairment, Verbal paraphasia	62	67	5	F	1050	T(m = i > s)≥F(o > c)>P	III	—	2	2	1	1	0	0	—
4	bvFTD	Disinhibition, Stereotypic behavior	64	74	10	M	950	F(l = c = o)=T(m = i > s)>P	III	—	2	—	—	—	0	0	—
5^b^	bvFTD	Stereotypic behavior	58^c^	67	9^c^	F	1120	T(i > s = m)≥F(l = c = o)>P	III	2	—	—	—	—	1	—	0
6^d^	bvFTD	Disinhibition	59	69	10	F	1020	F(l = c = o)≥T(m = i > s)	IV	3	—	2	0	1	0	0	1
7	PPA, Unspecified dementia	Unspecified aphasia, Memory impairment	—	66	—	M	1310	T(m = i > s)F(l = c > o)>P	IV	2	—	—	—	—	2	0	2
8	bvFTD	Disinhibition	59	64	5	M	1080	F(c = o > l)=T(m = i > s)>P	IV	3	—	2	1	0	0	0	3
9	bvFTD, PPA	Difficulty in using polite language	57	64	7	F	950	T(m = i > s)≥F(l = c = o)>P	IV	2	—	2	2	1	0	2	2
10^e^	bvFTD	Euphoria, Comprehension impairment	53	70	17	M	800	F(l = c = o)=T(m = i > s)	IV	3	3	—	—	—	—	—	2
11	Unspecified dementia	Alexithymia, Stereotypic behavior	41	51	10	F	530	F(l = c = o)≥T(m = i > s)>P	IV	—	3	2	1	3	2	2	—
12^f^	bvFTD	Disinhibition, Executive dysfunction	43	52	9	M	970	F(l = c = o)≥T(m = i > s)>P	—	3	3	2	2	2	2	2	3

*Note*: This table demonstrates the pathological distribution of phosphorylated tau‐positive structures, including both intraneuronal inclusions and degenerative neurites, and the clinical information of each PiD case. Mean age at onset was 54.3 years old, and mean disease duration between symptom onset and death was 8.2 years. Mean brain weight was 955 g.

Abbreviations: bvFTD, behavioral variant frontotemporal dementia; c, dACC; F, female; F, frontal lobe; l, dlPFC; i, inferior temporal gyrus; M, male; m, middle temporal gyrus; nfvPPA, nonfluent variant primary progressive aphasia; o, OFC; P, parietal lobe; PiD, Pick's disease; PPA, primary progressive aphasia. s, superior temporal gyrus; T, temporal lobe. Ordinal scores: 0—none to rare, 1—slightly, 2—moderate, 3—severe, (‐)—not done.

^a^ The pathological staging method used was based on a previous study.^17^ Phase I: Tau pathology is limited to the frontal lobe and limbic regions (the amygdala, entorhinal cortex, dentate gyrus of hippocampus, cornu ammonis of hippocampus, anterior cingulate gyrus, orbitofrontal cortex, middle frontal cortex, superior‐middle frontal cortex, and angular gyrus). Phase II: Tau pathology propagates to the primary sensory cortex, basal ganglia (the striatum and globus pallidus), thalamus, midbrain (the substantia nigra and red nucleus), pons (the locus coeruleus and raphe nucleus), dorsal nucleus of the vagus nerve, cerebellar dentate nucleus, and cervical spinal cord. Phase III: Tau pathology propagates to the primary motor cortex, and arcuate nucleus. Phase IV: Tau pathology propagates to the hypoglossal nucleus, visual cortex, cerebellar granular layer, and cerebral peduncle.

^b^ These cases were included in a previous study.^21^

^c^ Persecutory delusion preceded the emergence of cognitive decline by 10 years. Disease duration indicates the time since the onset of cognitive decline.

^d^ This case was reported in a previous study.^22^

^e^ This case was reported in a previous study.^23^ Secondary pathological feature is Lewy bodies in the brainstem.

^f^ Patient had MAPT mutation.

### Neuropathological findings in the present case

Postmortem examination revealed that the brain weighed 1090 g. Macroscopically, atrophy of both frontal lobes was evident (Figure 1a–c). The brain slices revealed slight atrophy of the hippocampus and entorhinal cortex (ENT) (Figure 1d,e). No obvious atrophy was observed in other regions.

**Figure 2:**
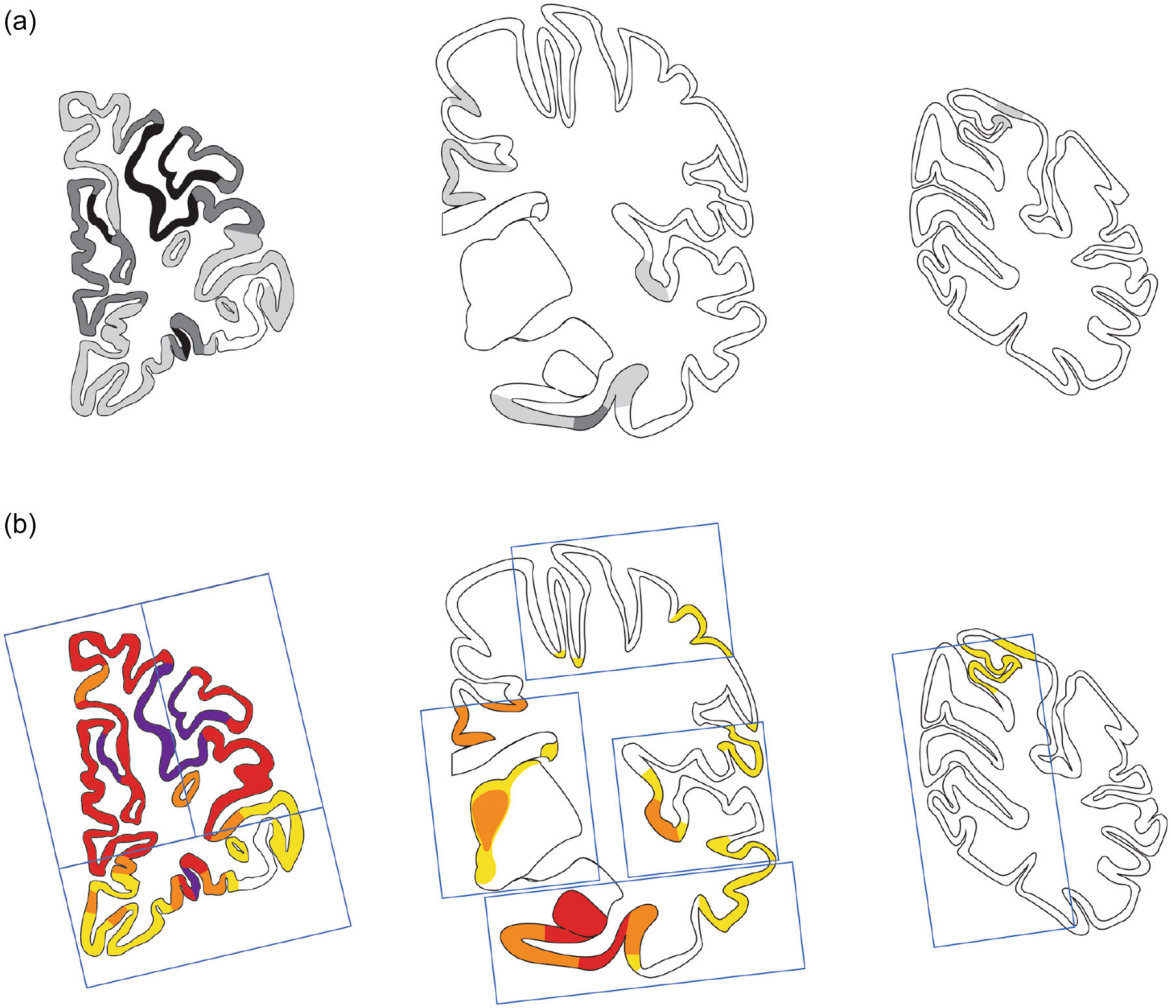
(a) Topographic distribution of cortical degeneration in present case (Case 1). The lesions were classified into three categories: slight (light gray), moderate (gray), and severe (black). Normal cerebral cortex is shown (white). Neuronal loss was prominent in severe lesions. (b) Topographic distribution of phosphorylated tau‐positive intraneuronal inclusions: Pick body in present case (Case 1). Lesions were classified into four categories: slight (yellow), moderate (orange), severe (red), most severe (purple). The lesions marked in purple indicate areas where phosphorylated tau‐positive intraneuronal inclusions were reduced due to significant neuronal loss.

**Figure 3:**
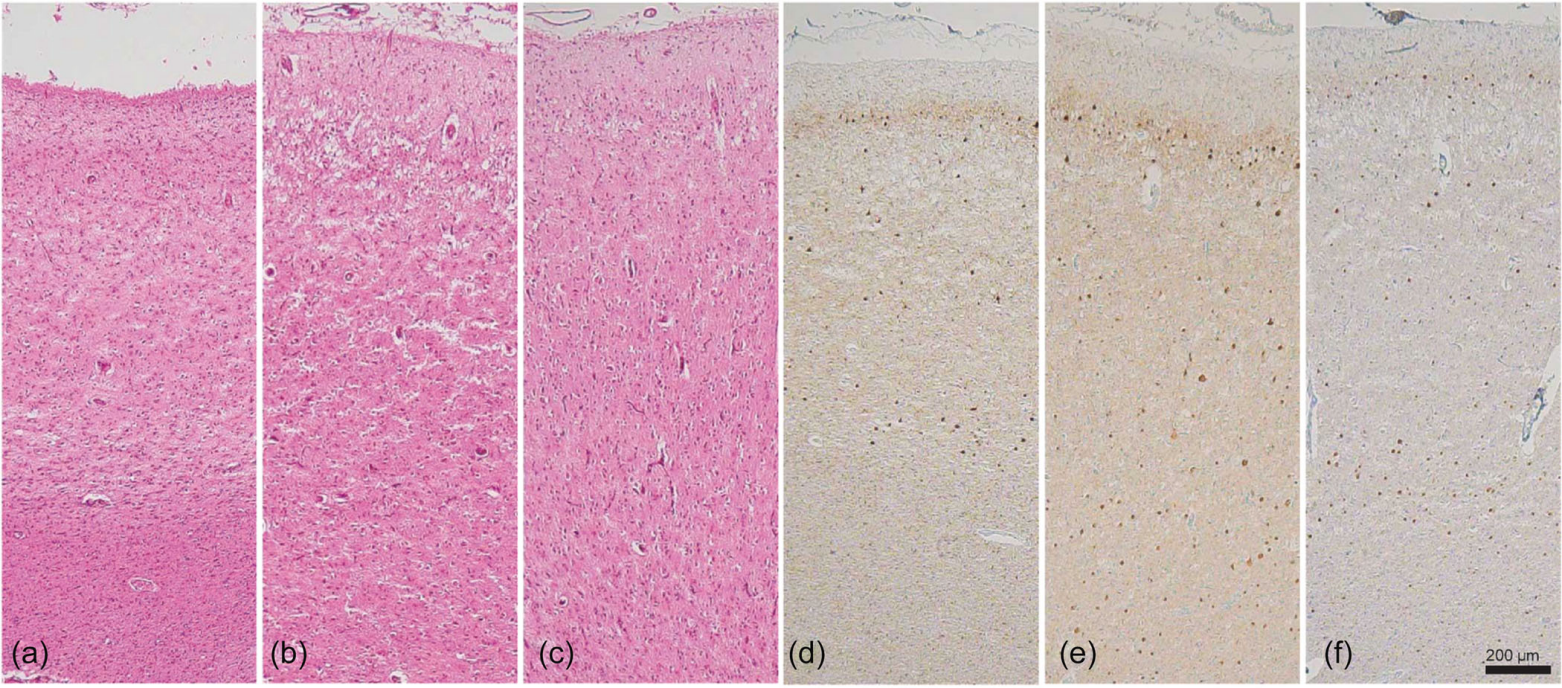
Microscopic findings in the frontal cortex. (a, d) Dorsolateral prefrontal cortex, (b, e) dorsal anterior cingulate cortex, (c, f) orbitofrontal cortex. (a–c) Hematoxylin and eosin (H&E) staining, (d–f) AT8. (a–c) H&E staining revealed neuronal loss and prominent spongiosis. (d–f) Phosphorylated tau‐positive intraneuronal inclusions were predominantly found in Layers II–III and V–VI.

Microscopically, the brain showed cortical degeneration in the frontal lobes, with predominance in the dorsolateral prefrontal cortex (dlPFC) and dorsal anterior cingulate cortex (dACC), as determined by H&E staining (Figures 2a, 3a–c). Neuronal loss was evident in dlPFC and dACC. Slight cortical degeneration was observed in ENT and the occipitotemporal cortex (OTC). H&E staining of the hippocampus revealed PBs in the cornu ammonis (CA) (Figure 1f). Bodian staining clearly showed PBs in the dentate gyrus (DG) and CA (Figure 1g,h). Holzer staining revealed gliosis in the cerebral white matter confined to the frontal lobe and medial temporal lobe. Cortical degeneration in the superior, middle, and inferior temporal gyri, parietal lobes, and occipital lobes were unremarkable. Betz's giant pyramidal cells in the precentral gyrus were preserved. In the medulla oblongata, KB staining revealed preserved luxol fast blue myelin staining in the pyramidal tracts. Secondary motor neurons in the anterior horn were preserved in the cervical spinal cord.

Immunohistochemical findings revealed AT8‐ and RD3‐positive, but RD4‐negative NCIs in DG, CA, and ENT (Figure 1i–k). Phosphorylated tau‐positive NCIs were found in the prefrontal cortex (PFC), predominantly in the dlPFC (Figure 3d), dACC (Figure 3e), and orbitofrontal cortex (OFC) (Figure 3f), as well as in the ENT, OTC, the middle and inferior temporal gyrus, insular gyrus, and superior parietal lobule (Figure 2b). In the neocortex of the frontal lobe, phosphorylated tau‐positive NCIs were predominantly observed in Layers II–III and V–VI (Figure 3d–e). NCIs were also found in the mediodorsal nucleus of the thalamus (MD), caudate nucleus, ventral tegmental area of the midbrain, oculomotor nucleus, reticular formation of the midbrain, periaqueductal gray, pontine nuclei, dorsal raphe nuclei, locus coeruleus, and anterior horn cells of the cervical spinal cord. Very few were found in the dorsal motor nucleus of the vagus and the postcentral gyrus. They were not observed in the calcarine sulcus, inferior olive nucleus, hypoglossal nucleus, cerebellar dentate nucleus, or granule cell layer. No TDP‐43, FUS, or α‐synuclein‐positive structures were found.

Based on these findings, this patient was pathologically diagnosed with FTLD‐tau with 3‐repeat tau: PiD. The area of degeneration in this case corresponded to phase II according to the staging of a previous study.^17^

### Clinical and neuropathological comparison with PiD case series in our institution

The demographic characteristics of the 12 patients with PiD in our institution are shown in Table 1. The mean disease durations by pathological phase were 1.5 years (Phase II), 7.8 years (Phase III), and 9.8 years (Phase IV). The mean disease duration according to clinical subtypes was 8.6 years for bvFTD and 6.3 years for those with language disorders. The present case is the only one in our institution that has been evaluated as Phase II. Of the PiD case series, eight (Cases 1, 2, 4, 6, 8, 10, 11, and 12) showed frontal‐lobe‐predominant degeneration, and four (Cases 3, 5, 7, and 9) showed temporal‐lobe‐predominant degeneration. Of the eight cases with frontal‐lobe‐predominant degeneration, six (Cases 1, 4, 6, 8, 10, and 12) had a clinical diagnosis of bvFTD, one (Case 1) presented with early symptoms of apathy, and six (Case 2, 4, 6, 8, 11, and 12) exhibited early symptoms of disinhibition or stereotypic behavior. Of the four cases with temporal lobe predominant degeneration, three (Cases 3, 7, and 9) were clinically diagnosed with aphasia.

Genetic studies, such as microtubule‐associated protein tau gene mutations, could not be performed because of a lack of frozen samples from the present case.

## DISCUSSION

Clinicopathological evaluation of PiD with a short disease duration, such as the present case of 1.5 years, is rare and important, as it potentially elucidates the association between clinical symptoms and pathological lesions.

### Clinicopathological features in the present case in relation to apathy or depression

Although the present case had been diagnosed as depressive disorder, the symptoms can be better interpreted as apathy, given that there was a marked decrease in activity and interest, including in himself, while the expression of distress was not conspicuous.^24^, ^25^

Severe degeneration of the dACC and lateral frontal lobe was observed. This finding is consistent with previous neuropathological^26^ and neuroradiological^27^ studies. In the present case, OFC was less degenerated than dlPFC and dACC. Since OFC is thought to be associated with behavioral inhibition,^28^ the distribution of lesions in this case (which consisted dominantly in the dlPFC and dACC in three cortical surfaces of the frontal lobe) was consistent with the clinical course, where apathy preceded disinhibition.

The patient had not been evaluated by any specific neuropsychological test, and this is a major limitation of this report.

### Neuropathological features of the present case as early PiD

In lesions with the most severe tissue degeneration and neuronal loss, the number of PBs tended to be lower than that in surrounding lesions with moderate degeneration. Beyond the areas with the strongest degeneration in the frontal lobe, more degenerated areas had a higher number of PBs. This suggests that the extracellularly released neuronal inclusion bodies in PiD due to neuronal death are less likely to remain in the tissue, unlike the ghost tangles observed in Alzheimer's disease.

A hypothesis has been proposed that aggregated proteins in neurodegenerative diseases propagate via association, projection and commissural fibers.^29^, ^30^, ^31^ In this case, the frontal lobe was strongly degenerated, and degeneration of the MD was evident, while degeneration of ventral lateral nucleus of the thalamus (VL) was less pronounced. Because the MD has reciprocal fiber connections with the frontal lobe,^32^ our findings are consistent with the hypothesis of neurodegenerative protein propagation via intraneuronal connections. In the MD, neuropil atrophy, and a mild increase in neuronal density compared to VL were found. Therefore, in the degeneration of the MD in this case, tau‐induced neurodegeneration and anterograde degeneration due to frontal lobe degeneration may have coexisted.

Degenerative lesions in the PFC were not in consecutive regions in a coronal slice of PFC. This suggests that the PFC, which showed the most significant pathological changes in the present case, may have had multiple lesion expansion “epicenters.”

### Clinicopathological features in PiD case series

Of the eight cases in this series (Table 1) with predominant PFC degeneration, the representative case (Case 1) was the only one with predominant degeneration of the dlPFC; in addition, only this case (Case 1) initially presented with apathy, while six cases (Cases 2, 4, 6, 8, 11, and 12) initially presented with either disinhibition or stereotypic behavior. One case (Case 10), presenting with euphoria and comprehension impairment as initial symptoms, exhibited predominant PFC degeneration. However, the specific pattern of degeneration within PFC was unclear. This may be due to the long duration of the disease in this case and the effects of advanced neurodegeneration. The absence of a clear trend toward more severe degeneration of OFC in cases exhibiting pronounced disinhibition and stereotypic behavior may be attributable to the same underlying cause.

A comparison of the case series suggests that, with a long‐term disease course, discontinuous areas of severe degeneration in PFC may progressively merge into more severe and continuous areas of degeneration. Consequently, in PiD cases at an advanced phase of neurodegeneration, identifying specific sites of predominant degeneration within PFC becomes challenging. Therefore, the representative case is distinctive in comparison with other cases in the series, as it demonstrates an association between degenerative areas and clinical symptoms predominantly characterized by apathy.

Disease duration in PiD cases at our institution did not always correlate with the pathological phase (Table 1), which is consistent with previous studies.^17^, ^33^ The onset of dementia in patients with neurodegenerative diseases depends on social situations, such as whether people around the patient can notice their behavioral changes.

In our case series, the pathological structures in the substantia nigra and locus coeruleus tended to be more severe in cases with broadly propagated pathological structures in other lesions, which is consistent with a previous study.^17^

In Case 1, the deposits of phosphorylated tau‐positive NCIs in the middle temporal gyrus were clear, while the degeneration of the superior temporal gyrus was minor. This suggests that primary sensory areas, excluding the olfactory cortex, are less vulnerable to PiD degeneration.

Our case series did not replicate previous findings^33^ that language variant patients have a longer prognosis than bvFTD patients. This may be due to the limited number of patients with language disorders in our study.

## CONCLUSION

Previous studies have indicated that apathy is associated with dACC and dlPFC, while disinhibition and stereotypic behavior are associated with OFC. The clinical symptoms in this case, which were mainly apathy and fewer signs of behavioral variant symptoms, were consistent with the pathological lesion, which was mainly in the dlPFC. This suggests that PFC lesions remain localized in the early stages of PiD, and that the early clinical manifestations may be associated with the functional impairment caused by the focal degenerative lesion. On the other hand, the comparison between the representative case and the case series suggests that, in advanced PiD cases, identifying specific patterns of PFC degeneration is challenging because, as PiD progresses, focal degenerative areas in the PFC may progress into consecutive and more severe degenerative lesions.

Further cases need to be accumulated to elucidate the pathogenesis of PiD.

## AUTHOR CONTRIBUTIONS

Araki Kimura performed microscopy analysis, data analysis, and drafted the manuscript. Ito Kawakami and Kenji Ikeda participated in the study design and microscopy analysis. Akito Nagakura, Kazuhiro Niizato, and Kenichi Oshima organized the brain archives and selected appropriate cases. Ito Kawakami, Kenji Ikeda, and Tadafumi Kato provided helpful advice on data interpretation and elaborated on the manuscript. Masato Hasegawa supervised study coordination. All the authors have read and approved the final version of the manuscript.

## CONFLICT OF INTEREST STATEMENT

The authors declare no conflicts of interest.

### ETHICS APPROVAL STATEMENT

Personal information of the patients and their bereaved families was carefully protected. The Ethics Committee of the Tokyo Metropolitan Matsuzawa Hospital approved the use of this database. The study was conducted in accordance with the ethical standards laid down in the 1964 Declaration of Helsinki and its later amendments.

## PATIENT CONSENT STATEMENT

After the patient's death, consent for autopsy and postmortem analysis for research purposes was obtained from the next of kin.

## CLINICAL TRIAL REGISTRATION

N/A.

## Data Availability

The data supporting this study are available from the corresponding author upon reasonable request.
